# Optimization and Decision Science

**DOI:** 10.1155/2015/159169

**Published:** 2015-03-25

**Authors:** F. Hosseinzadeh Lotfi, A. Amirteimoori, Barnett Parker, Mohammad Khodabakhshi, Jie Wu, M. Vaez-ghasemi

**Affiliations:** ^1^Department of Mathematics, Islamic Azad University, Science and Research Branch, Tehran, Iran; ^2^Department of Mathematics, Islamic Azad University, Rasht Branch, Rasht, Iran; ^3^Department of Health Administration, Pfeiffer University, Charlotte, USA; ^4^Department of Mathematics, Shahid Beheshti University, Tehran, Iran; ^5^Department of Management Science, University of Science and Technology of China, Hefei, Anhui, China

## 1. Introduction

Operational research (OR) includes a wide range of problem-solving techniques and methods applied in the pursuit of improved decision-making and efficiency. OR involves the construction of mathematical models that attempt to describe the system. Because of the computational and statistical nature of most of these fields, OR also has strong ties to computer science and analytic. Operational researchers faced with a new problem must determine which of these techniques are most appropriate given the nature of the system, the goals for improvement, and constraints on time and computing power. The main focus of this special issue will be on the new research ideas and results for the OR. Considering the wide applications of OR, it seems natural that this journal selected it as the theme of its special issue.

## 2. Overview

This special issue is composed of ten research papers; the covered subjects include topics such as ranking, congestion, fuzzy numbers and system, supply chain, and optimization problems. Below, a very brief overview of the featured works is given.

M. Rostami-Malkhalifeh et al. in their paper deal with evaluating congestion in free disposal hull (FDH) models. In the DEA literature, there exist many methods dealing with theory and application of congestion in convex technologies. Considering nonconvex technologies, this paper discusses congestion in free disposal hull. The presented method shows the sources of congestion and estimates its amounts and also detects the losses amounts of output due to congestion. In a paper by M. Fallah and A. Mohajeri, green supply chain management is considered. Nowadays, due to global warming and climate changes, it is an important issue. Here, the authors formulate a comprehensive closed-loop model for the logistics planning considering profitability and ecological goals. They noted that the profitability criterion supported the cyclic network with the minimum costs and maximum service level. In the work provided by M. Rostami-Malkhalifeh et al., a new method is presented for solving fuzzy system of linear equations with crisp coefficients matrix and fuzzy or interval right hand side. The authors derived conditions for the existence of a fuzzy or interval solution of linear system. A work by M. Rostami-Malkhalifeh et al. discusses calculating superefficiency and then ranking the units based on it. The authors noted that most of the existing models do not provide the projection of Pareto efficiency. Thus, they introduced a model based on which the projection of Pareto-efficient can be achieved. The presented model is unit invariant and feasible and makes the amount of inefficiency effective in ranking. In a paper by M. Rostami-Malkhalifeh et al., a procedure is provided for ranking DMUs in DEA technique considering ideal and anti-ideal points. They formulated a model which is introduced to compute the performance of units for these two points through using common set of weights. The important feature of this model is that it can rank all DMUs by solving only three programs and it can rank all the extreme and nonextreme efficient DMUs. In the work by L. G. Moazam and T. Allahviranloo, a new concept of the 2nd power of a fuzzy number is introduced. The authors noted that this method is exponent to production (EP) method that provides an analytical and approximate solution for fully fuzzy quadratic equation (FFQE). The work by M. Rostami-Malkhalifeh et al. studies the inverse DEA technique while the nonradial enhanced Russell model is being used. They also introduced necessary and sufficient conditions for extra inputs or lack outputs determination and also investigated for showing the extra input or lack output. In the work by M. R. Abu Bakar et al., a new method is provided for the directional slack-based measure for the inverse DEA. The authors noted that the presented method elucidates the inverse directional slack-based measure model within a new production possibility set and noted that this approach was investigated in this study with reference to a resource allocation problem. It is possible to simultaneously consider any alterations of certain outputs associated with the efficient decision-making unit. In the paper by S. Kim, it is mentioned that gamification means the use of various elements of game design in nongame contexts including workplace collaboration, marketing, education, military, and medical services. This paper suggested the decision criteria for selection of gamification platform to support a systematic decision-making process for managements. They noted that the criteria are derived from previous works on gamification, introduction of information systems, and analytic hierarchy process.

A. Grilo and J. Santos noted that there exists a shortage in the literature regarding the efficiency evaluation and productivity evolution of the new technology-based firms (NTBFs) in the incubation scope. Thus, they provided a model considering DEA analysis in order to incubate NTBFs for assessment and improvement in the efficiency. Moreover, they also used Malmquist index.

*F. Hosseinzadeh Lotfi* *F. Hosseinzadeh Lotfi* *A. Amirteimoori* *A. Amirteimoori* *Barnett Parker* *Barnett Parker* *Mohammad Khodabakhshi* *Mohammad Khodabakhshi* *Jie Wu* *Jie Wu* *M. Vaez-ghasemi* *M. Vaez-ghasemi*

